# An Efficient Catalytic DNA that Cleaves L-RNA

**DOI:** 10.1371/journal.pone.0126402

**Published:** 2015-05-06

**Authors:** Kha Tram, Jiaji Xia, Rachel Gysbers, Yingfu Li

**Affiliations:** 1 Department of Chemistry and Chemical Biology, McMaster University, Hamilton, Ontario, Canada; 2 Department of Biochemistry and Biomedical Sciences, McMaster University, Hamilton, Ontario, Canada; 3 Department of Biochemistry and Biomedical Sciences and Origins Institute, McMaster University, Hamilton, Ontario, Canada; 4 Department of Biochemistry and Biomedical Sciences, Department of Chemistry and Chemical Biology, and Origins Institute, McMaster University, Hamilton, Ontario, Canada; The University of Queensland, AUSTRALIA

## Abstract

Many DNAzymes have been isolated from synthetic DNA pools to cleave natural RNA (D-RNA) substrates and some have been utilized for the design of aptazyme biosensors for bioanalytical applications. Even though these biosensors perform well in simple sample matrices, they do not function effectively in complex biological samples due to ubiquitous RNases that can efficiently cleave D-RNA substrates. To overcome this issue, we set out to develop DNAzymes that cleave L-RNA, the enantiomer of D-RNA, which is known to be completely resistant to RNases. Through *in vitro* selection we isolated three L-RNA-cleaving DNAzymes from a random-sequence DNA pool. The most active DNAzyme exhibits a catalytic rate constant ~3 min^-1^ and has a structure that contains a kissing loop, a structural motif that has never been observed with D-RNA-cleaving DNAzymes. Furthermore we have used this DNAzyme and a well-known ATP-binding DNA aptamer to construct an aptazyme sensor and demonstrated that this biosensor can achieve ATP detection in biological samples that contain RNases. The current work lays the foundation for exploring RNA-cleaving DNAzymes for engineering biosensors that are compatible with complex biological samples.

## Introduction

Catalytic DNAs or DNAzymes are single-stranded DNA molecules that can catalyse a chemical reaction. They are isolated from a random-sequence DNA library using an established technique known as “*in vitro* selection” [1,2]. The first example of a catalytic DNA was provided by Breaker and Joyce in 1994 with a Pb^2+^-dependent RNA-cleaving DNAzyme [3]. Since then, many DNAzymes have been discovered for an impressive range of chemical reactions [4–10]. However, RNA-cleaving DNAzymes have been widely examined for several reasons. First, the transesterification reaction involving RNA is well known in biology as many protein enzymes and ribozymes rely on this chemistry to cleave RNA [11–14]. This creates a unique opportunity to compare catalytic abilities of DNA, RNA and protein for the same reaction. Second, the historical precedency of RNA-cleaving DNAzymes has directed the research community to use RNA cleavage as a model reaction to study DNA-based catalysis. In addition, many RNA molecules are medically important and thus RNA-cleaving agents including RNA-cleaving DNAzymes have the potential to be developed into therapeutic agents [15,16]. Moreover, the existence of efficient RNA-cleaving DNAzymes [15–19] and effective strategies for engineering stimuli-responsive RNA-cleaving DNAzymes makes this catalytic system a favoured choice for biosensor engineering [20–34].

A number of RNA-cleaving DNAzyme biosensors have been reported and applied for the detection of many different analytes [20–34]. However, most of these sensors have been designed to function in simple sample matrices and are not fully functional in complex biological samples. This is because the RNA substrate for these DNAzymes is in the natural D-configuration (D-RNA), which is also the substrate of natural RNases that are pervasive in biological samples. Since RNases are powerful enzymes that cleave D-RNA more efficiently than DNAzymes, biosensors engineered with D-RNA-cleaving DNAzymes are prone to producing false-positive signals with biological samples. This drawback has significantly restricted the application of these biosensors, calling for the development of RNase-resistant RNA-cleaving DNAzymes that are both efficient and compatible with biological samples.

With this motivation in mind, we set out to develop DNAzymes that cleave L-RNA, the enantiomer of D-RNA. Since L-RNA is known to be highly resistant to RNase degradation [35], replacing D-RNA with L-RNA offers an attractive solution for taking advantage of RNA-cleaving DNAzymes for biosensing applications. The use of L-RNA as the cleavage site, however, can post a challenge for DNAzyme isolation as it has been shown that the enantiomeric difference between L- and D-isomers prevents extensive Watson-Crick base-paring interactions [36,37], which are often employed by RNA-cleaving DNAzymes for substrate binding. On the other hand, DNAzymes for biosensing applications almost exclusively involve the use of a chimeric DNA/RNA substrate that contains a single ribonucleotide as the cleavage site, a strategy we will also adopt in this study. The use of the single L-RNA moiety should minimize the impact on the DNAzyme-substrate interactions. In addition, it has been shown that D and L stereoisomers are still capable of engaging each other through alternative interactions [38]. More importantly, the Joyce group set precedence in 2002 by successfully isolating an L-RNA-cleaving DNAzyme from a random-sequence DNA pool [39]. However, the reported DNAzyme exhibits a catalytic rate constant of merely 0.001 min^-1^, several orders of magnitude inferior than the best-known D-RNA-cleaving DNAzymes (with rate constants as high as 10 min^-1^ [4]). The reduced efficiency makes this DNAzyme unsuitable for biosensor engineering. Therefore, the key objective of our study is to develop a significantly more active L-RNA-cleaving DNAzyme.

Herein we report an L-RNA-cleaving DNAzyme that exhibits a catalytic rate constant of ~3 min^-1^. We have also used this DNAzyme and a well-known ATP-binding DNA aptamer to construct a ligand-responsive biosensor. Finally we have demonstrated that this biosensor can achieve ATP detection in biological samples that contain RNases.

## Results and Discussion

### *In vitro* selection

**Fig 1:**
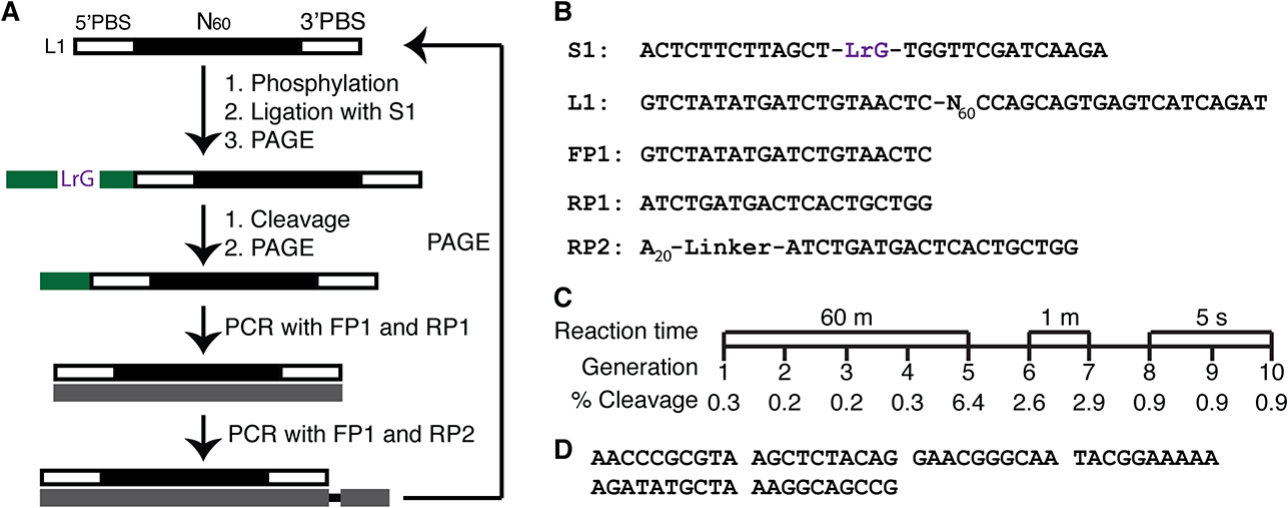
*In vitro* selection of L-RNA-cleaving DNAzymes. (A) *In vitro* selection scheme. (B) The sequences of the substrate (S1), DNA library (L1) and PCR primers (FP1, RP1 and RP2) used during *in vitro* selection. (C) The reaction time and percentage cleavage for each selection cycle. (D) The sequence of LRD-B, which is featured in this study (only the 60-nt random domain is shown).

A pool of 10^14^ molecules, denoted L1 and made of 60-nt (nt: nucleotide) random domain flanked by two 20-nt primer-binding sites, was used for the *in vitro* selection experiment. The *in vitro* selection scheme is shown in Fig 1A. L1 was first phosphorylated and ligated to a chimeric DNA/RNA substrate S1 that contains a single guanosine L-ribonucleotide (LrG) as the cleavage site (Fig 1B). Upon purification using denaturing gel electrophoresis (dPAGE), the ligated construct was incubated for 60 minutes in the selection buffer that contains Mg^2+^ and Mn^2+^ as divalent metal ion cofactors (for the consideration that most RNA-cleaving DNAzymes require divalent metal ions for high catalytic activity). The cleavage product was purified by dPAGE and subjected to two polymerase chain reactions (PCR1 and PCR2). PCR1 used two standard DNA primers, forward primer FP1 and reverse primer RP1 (Fig 1B); however PCR2 used FP1 and RP2, which contains an A20 tail separated by a non-amplifiable linker (Fig 1B). Therefore, PCR2 produced two DNA strands with unequal sizes, which permit the separation of the coding strand by dPAGE. The purified DNA amplicon was used as the DNA pool for the next cycle of selective enrichment. After 5 rounds, a cleavage signal was observed. In order to isolate the most efficient DNAzymes, the RNA cleavage time was reduced to 1 min for rounds 6 and 7, and then to 5 sec for rounds 8–10 (Fig 1C). The round-10 DNA pool was cloned and sequenced.

The top three clones are denoted LRD-A, LRD-B, and LRD-C (S1 Fig). All three classes were capable of cleaving the attached S1 substrate. It was found that LRD-B (first-order rate constant = 0.34 ± 0.05 min^-1^) was slightly more active than LRD-A (0.33 ± 0.04 min^-1^) whereas the catalytic rate of LRD-C was much lower (0.04 ± 0.01 min^-1^, S2 Fig). We chose LRD-B for further study.

### Deletion walking experiment

**Fig 2:**
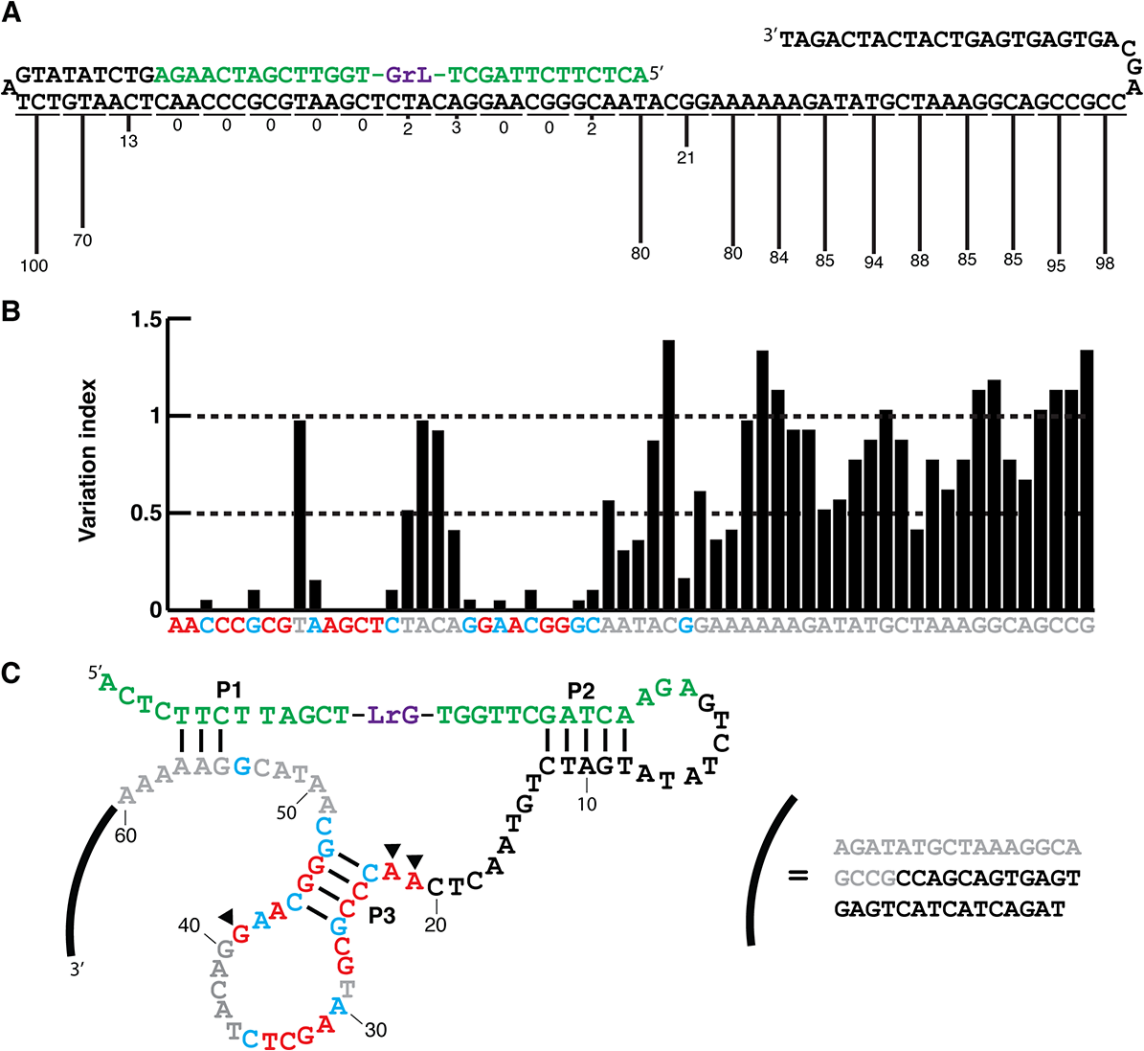
Characterization of LRD-B. (A) Deletion walking experiment. Starting from the 3′-end of LRD-B, sets of three nucleotides were sequentially removed to determine sites that contain important structural and catalytic residues. The numbers represent the normalized activity against untruncated sequence (taken as 100). (B) Reselection with the mutant library of LRD-B. Nucleotides labeled in red are absolutely conserved, blue are highly conserved, and grey are nucleotides that are non-conserved. (C) The proposed secondary structure of LRD-B. Solid triangles indicate nucleotides that show significant methylation interference.

To identify the catalytic core of LRD-B, a deletion walking experiment was conducted where groups of three nucleotides were removed one by one, starting from the 3′-end (Fig 2A). Each mutant was examined to determine if each 3-nucleotide deletion would affect the catalytic activity. The removal of any functionally important nucleotide is expected to affect the performance of the DNAzyme, and the severity of disruption can be measured through the reduction of the RNA cleavage activity, measured as the relative activity normalized against the full-length LRD-B. The deletion walking experiment indicates that the first half of the sequence of the LRD-B is critical for the DNAzyme function whereas the second half is dispensable.

### Re-selection with a mutagenized LRD-B DNAzyme pool

A reselection experiment was then conducted with a partially randomized library of LRD-B in which each nucleotide in the original random-sequence domain was chemically synthesized to have a degeneracy of 30% (70% for the wild-type nucleotide and 10% each for the remaining three nucleotides). After six rounds of selective amplification, the DNA pool was sequenced and individual sequences were analyzed to identify conserved nucleotides. Each individual nucleotide was organized using a variation index (VI) using a method we developed previously [40]. Nucleotides that were absolutely conserved were coded in red (VI = 0), important residues in blue (VI < 0.25) and not important in grey (VI > 0.25) (Fig 2B) [40]. Nucleotides with VI of 0 do not tolerate any base substitution; they either participate directly in catalysis or are essential for structural formation. Blue-coded nucleotides can tolerate some level of mutation; it is possible that these nucleotides play a significant role in structural organization rather than catalysis. In contrast, nucleotides coded in grey are not functionally important. Based on sequence analysis and results obtained from the truncation and reselection experiments, we proposed a simple structure as illustrated in Fig 2C.

### Methylation with dimethyl sulphate

Initial assessment of LRD-B’s secondary structure reveals a simple internal stem-loop that may be highly important since many residues in the loop are highly conserved (Fig 2C). Since several highly conserved nucleotides are adenine and guanine nucleotides, methylation reaction with dimethyl sulphate (DMS) was then carried out to further probe their importance to the function of the DNAzyme. DMS can methylate N-7 of guanine and N-3 of adenine and placing a methyl group on nitrogen atoms of functionally important guanines and adenines is expected to interfere the catalytic function of the DNAzyme. These nucleobases will appear as undermethylated (protected against methylation in this assay) [40]. As revealed in S3 Fig, A21, A22, and G41 were observed to have the most significant interference with methylation.

### Design of a *trans*-acting LRD-B construct

**Fig 3:**
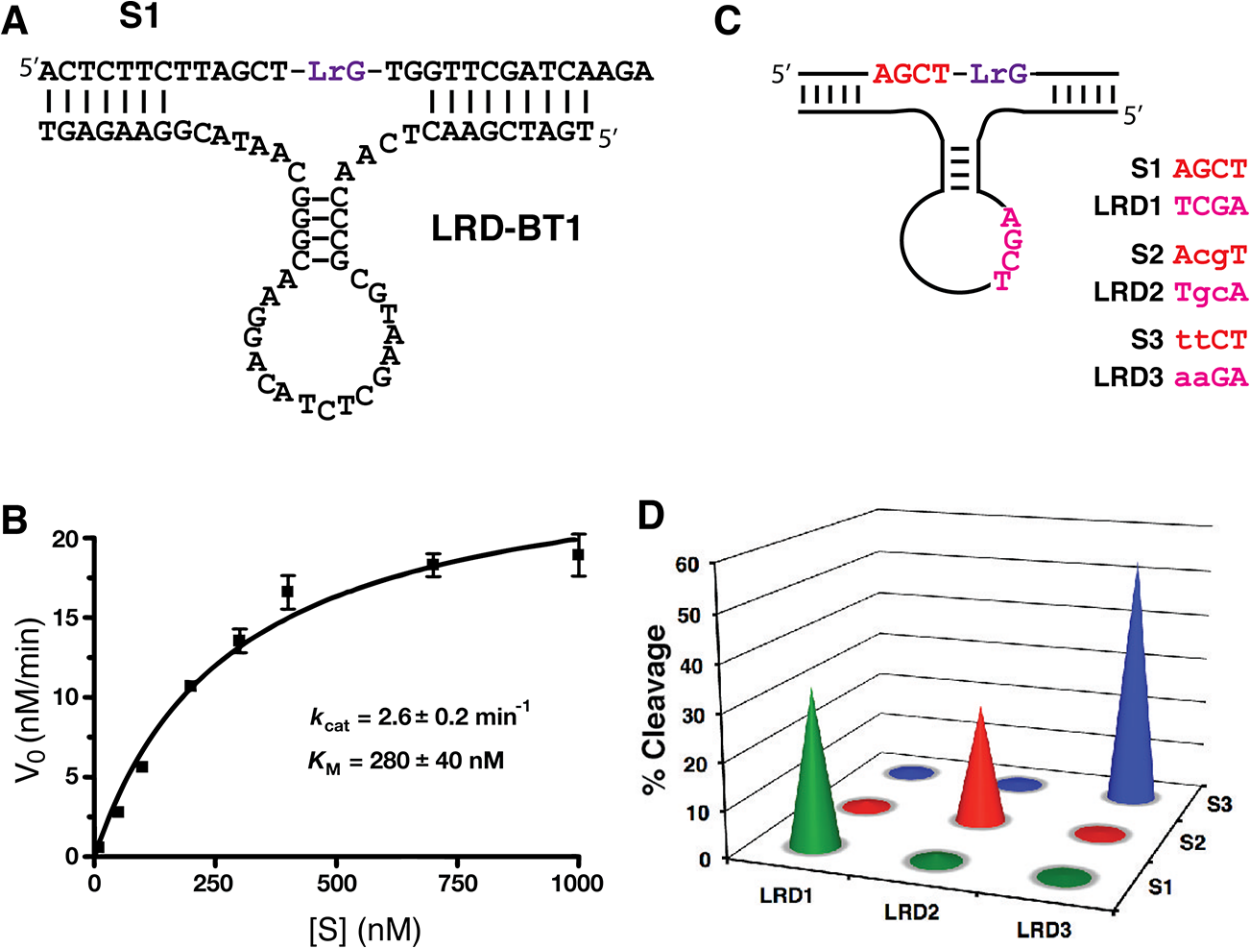
A *trans*-acting DNAzyme construct. (A) Optimized *trans*-acting sequence denoted as LRD-BT1. (B) Multiple-turnover kinetic analysis of LRD-BT1. The initial velocity is plotted against substrate concentrations and the data is fitted to the Michaelis-Menten equation v = *k*_cat_[S]/(*K*_M_ + [S]). (C) Identification and confirmation of a kissing loop. Three substrates (S1, S2 and, S3) and their matched DNAzymes (LRD-BT1, LRD-BT2, and LRD-BT3) were tested for the cleavage activity. The lowercase letters denote altered nucleotides.

Once we have identified the key nucleotides that are important to the function of LRD-B, we sought to convert the *cis*-acting catalyst into a *trans*-acting enzyme. The primer binding sites and some non-conserved nucleotides were first removed from the sequence of LRD-B. Additional nucleotides were also introduced to create stronger binding arms between the substrate strand and the DNAzyme strand. The final *trans*-acting LRD-B (denoted LRD-BT1) is shown in Fig 3A. The catalytic activity of LRD-BT1 was assessed under multiple turnover conditions. LRD-BT1 was found to have *k*_cat_ of 2.6 ± 0.2 min^-1^ and a *K*_M_ of 280 ± 40 nM.

### Identification of a kissing loop in the DNAzyme structure

The secondary structure model for LRD-BT1 contains four absolutely conserved nucleotides—AGCT-in the loop of the hairpin. Our mutational analysis indicates that base alteration to any nucleotide in this motif results in complete loss of the catalytic activity (data not shown). Upon close inspection, we noticed another AGCT motif located immediately upstream of LrG cleavage site. This observation led us to speculate that these two motifs form a kissing loop, which might be important for the function of LRD-B. We believe that this would bring catalytically important nucleotides from the stem-loop closer to the cleavage site (Fig 3C).

To confirm the existence of the kissing loop and its importance to the function of LRD-BT1, we carried out compensation mutagenesis analysis. In this experiment, the introduction of base mutations to disrupt the kissing loop formation is expected to significantly diminish the catalytic activity while co-variations to restore the base-pairing interactions should also revive the catalyst. Two new enzyme-substrate pairs, LRD-BT2/S2 and LRD-BT3/S3, were constructed and all 9 enzyme-substrate combinations were examined for cleavage activity (Fig 3C). The results provided in Fig 3D show that each enzyme could only cleave its matching substrate, indicating that the kissing loop between the enzyme and the substrate strands does exist and is essential to the function of the DNAzyme.

### Substrate specificity and metal-ion dependency

**Fig 4:**
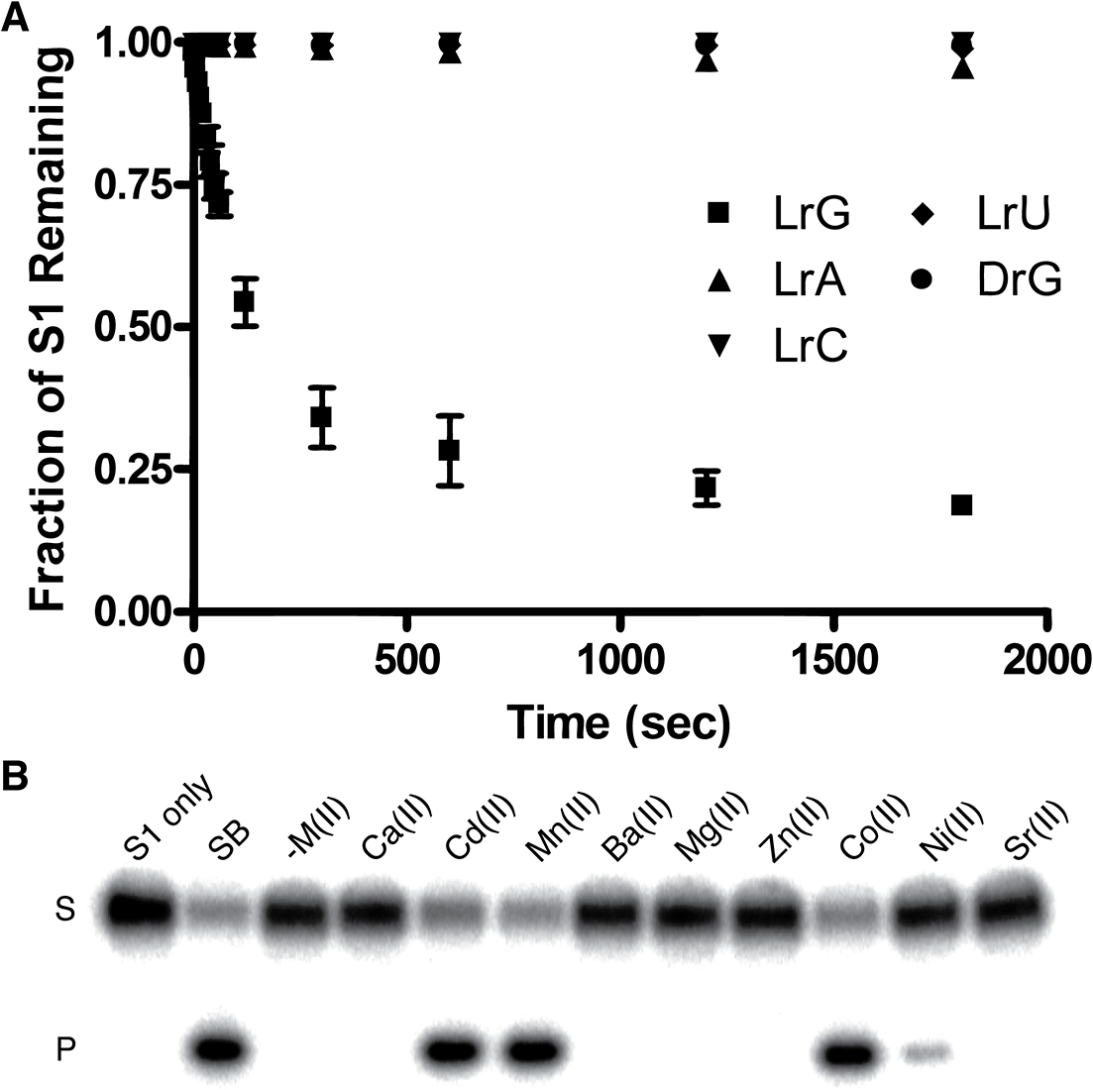
Further characterization of LRD-BT1. (A) Cleavage-junction selectivity. Cleavage profile of LRD-BT1 towards four substrates differing only at the cleavage site: LrG, LrA, LrC, LrU or DrG. (B) Metal-ion dependency. Reaction buffers contain 60 mM HEPES, pH 7.5, 300 mM NaCl, and 100 mM KCl, in addition to a divalent metal ion (15 mM) specified in the figure.

As the *in vitro* selection experiment used the substrate containing guanosine L-ribonucleotide (LrG) as the cleavage site, we were interested to see if LRD-BT1 can cleave other L-ribonucleotides including LrA, LrC, and LrU. In addition, we also tested the cleavage activity of LRD-BT1 towards the natural D-ribonucleotide. The data in Fig 4A indicates that LRD-BT1 has extremely weak activity towards all of these substrates.

We also evaluated the divalent metal ion requirement of LRD-B. We found that this DNAzyme exhibits robust activity in the presence of Mn^2+^, Cd^2+^, and Co^2+^, reduced activity with Ni^2+^, but is inactive in the presence Mg^2+^ (Fig 4B). Even though the original selection buffer contained both Mg^2+^ and Mn^2+^, LRD-B recruited Mn^2+^ as the divalent metal ion cofactors over Mg^2+^. This finding is not surprising given the fact that many previous studies have also shown that divalent transition metal ions are often the preferred divalent metal ions for DNAzyme mediated catalysis [3, 19, 41–44].

### Engineering a ligand-responsive LRD

**Fig 5:**
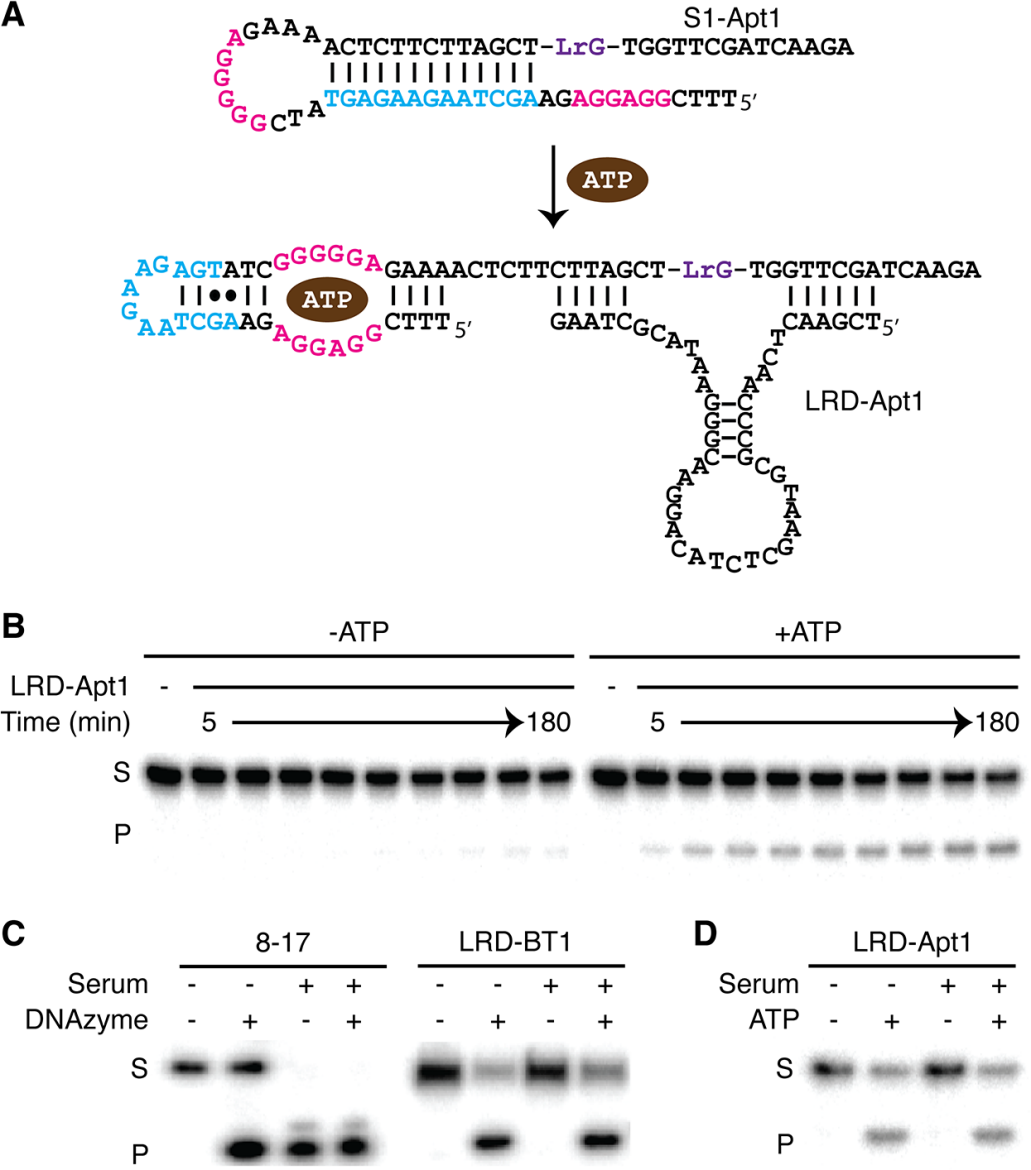
An ATP-responsive aptazyme constructed from LRD-BT1. (A) Sequence design and detection mechanism. Residues highlighted in red is the binding site for ATP, blue are DNAzyme-blocking nucleotides. (B) Time-dependent cleavage of S1-Apt1 by LRD-Apt1 in the absence and presence of 1 mM ATP. (C) Cleavage activity comparison of D-RNA and L-RNA systems in 75% human serum. (D) The functionality of the ATP-responsive aptazyme constructed with LRD-Apt1 in 75% human serum. S: substrate; P: cleavage product.

RNA-cleaving DNAzymes can be combined with aptamers for the engineering of allosteric DNAzymes or aptazymes [42, 45–47]. To derive an aptamer from LRD-B, we adopted a non-classical design previously reported by our group [30]. This approach uses an aptamer-containing substrate strand (S1-Apt1) with a sequence design in which part of the aptamer engages part of the substrate into a hairpin structure that prevents the access of the substrate by the DNAzyme. However, in the presence of the cognate ligand for the aptamer, the hairpin structure gives the way to the formation of ligand-aptamer complex, making the substrate fully accessible to the DNAzyme. We used the widely examined ATP-binding DNA aptamer [48] in the design of the aptazyme; the sequence design and the switching mechanism are illustrated in Fig 5A. As shown in Fig 5B, the cleavage of S1-Apt1 by LRD-BT1 (denoted LRD-Apt1) was found to be dependent on the presence of ATP in the time-dependent manner (also see S4 Fig).

### Stability and functionality of LRD in human serum

Our key motivation for isolating an L-RNA-cleaving DNAzyme is to develop an RNA-cleaving DNAzyme system that enables biosensing applications with RNase-containing biological samples. We first illustrated the RNase resistance capabilities of L-RNA versus D-RNA by treating L-RNA and D-RNA containing substrates with RNase T1 or RNase I. We found the L-RNA substrate remained fully intact while the D-RNA substrate was cleaved by the RNases (see S5 Fig). We then demonstrated the advantage of L-RNA-cleaving DNAzymes over the D-RNA-cleaving counterparts, we compared the stability of LRD-BT1 and 8–17 (a representative D-RNA-cleaving DNAzyme [15, 49]) in human serum. As shown in Fig 5C, although in the clean reaction buffer the cleavage of D-RNA substrate was dependent on the presence of 8–17, this dependence vanished in serum. Therefore, it is clear that any biosensor built with 8–17 will unavoidably lead to a false-positive signal in RNase-containing biological samples. In sharp contrast, the cleavage of L-RNA substrate is totally dependent on LRD-BT1 regardless of whether the reaction was performed in the pure reaction buffer or in human serum (Fig 5C). Similarly, the biosensor designed with LRD-BT1 for ATP detection was indeed able to develop ATP in complex sample matrix like human serum (Fig 5D).

## Conclusions

We have shown in this work that efficient DNAzymes can be isolated from a synthetic single-stranded DNA pool to cleave RNA in its unnatural L-configuration using the test-tube selection approach. We have examined the catalytic and structural properties of the best performing L-RNA-cleaving DNAzyme, LRD-B, and discovered that this catalytic DNA exhibits a first-order rate constant approaching 3 min^-1^, ~2500-fold more active than the previously reported L-RNA-cleaving DNAzyme. LRD-B is on a par with the best D-RNA-cleaving DNAzymes, suggesting DNA has a similar ability to cleave D and L RNA. Structurally LRD-B contains a kissing loop that is essential to the catalytic function. To the best our knowledge, such a structural motif has not been found in the structure of any D-RNA-cleaving DNAzyme. Since the kissing loop is formed between four nucleotides next to the cleavage site in the substrate strand and four nucleotides that are surrounded by several highly conserved nucleotides in the DNAzyme strand, we speculate that this interaction defines the catalytic core and serves to position catalytically important nucleotides in the DNAzyme next to L-RNA unit in the substrate. We have also converted the L-RNA-cleaving DNAzyme to a ligand-responsive sensor, taking advantage of an existing DNA aptamer that binds ATP, and have shown that this aptazyme is fully functional in biological samples that contain RNases. Taken together, the current work sets the stage for exploring RNA-cleaving DNAzymes for engineering biosensors that are compatible with complex biological samples.

## Materials and Methods

### Enzymes, chemicals and oligonucleotides

T4 DNA ligase, T4 polynucleotide kinase (PNK), ATP, and dNTPs were obtained from Thermo Scientific. Tth DNA polymerase was purchased from Biotools B & M Labs (Madrid, Spain). RNase I and RNase T1 were purchased from Epicenter (Madison, Wisconsin). [γ-^32^P]ATP and [α-^32^P]dGTP were from Perkin Elmer (Woodbridge, Ontario). Triethylamine trihydrofluoride (TEA-THF), dimethyl sulfate (DMS), piperidine, anhydrous dimethyl sulfoxide (DMSO) were purchased from Sigma-Aldrich (Oakville, Ontario). All other chemicals were purchased from BioBasic (Markham, Ontario) and used without further purification. Water used in this study was double-deionized and autoclaved.

Standard oligonucleotides were synthesized by solid-phase synthesis from Integrated DNA Technologies (Coralville, Iowa) while modified L-RNA oligonucleotides were obtained from Keck Biotechnology Resource Laboratory, Yale University (New Haven, Connecticut). All oligonucleotides were purified by 10% denaturing polyacrylamide gel electrophoresis (dPAGE) prior to use.

The 2′-O-TBDMS (tert-butyldimethylsilyl) protecting group of L-RNA was deprotected by dissolving the dry pellet in 100 μL of anhydrous DMSO and 125 μL of TEA-THF. This was followed by an overnight incubation at 60°C. To this mixture, 25 μL of NaOAc (3 M, pH 5.2) and 1 mL of butanol were added. The solution was stored at -20°C for 30 min and then centrifuged for 20 min at 15,000 g. The pellet was washed 3 times with 70% ethanol and purified by 10% dPAGE.

### *In vitro* selection with the initial library L1

One thousand pmol of L1 were used as the initial library. The DNA molecules in this pool were first labeled with ^32^P at the 5′-end in the presence of 10 units (U) of T4 PNK, 10 μCi of [γ-^32^P]ATP, and 1× T4 PNK buffer A (using the 10× buffer supplied by the vendor) for 20 min at 37°C in a reaction volume of 100 μL. This was followed by the addition of non-radioactive ATP to a final concentration of 1 mM and further incubation at 37°C to ensure complete phosphorylation. The reaction was stopped by heating at 90°C for 5 min. Upon cooling to room temperature (~23°C), 1000 pmol of S1 and 1000 pmol of T1 were added (T1 is the template for ligation and its sequence is 5′-TCATA TAGAC TCTTG ATCGA; the sequences of all other DNA molecules can be found in Fig 1). The reaction mixture was then heated to 90°C for 1 min and cooled to room temperature. Ten units of T4 DNA ligase and 25 μL of 10× T4 DNA ligase buffer (supplied by the vendor) were added to the reaction mixture (total reaction volume: 250 μL). The ligation reaction was carried out at room temperature for 2 h. The DNA in the mixture was precipitated by ethanol and the ligated product was purified by 10% dPAGE.

The purified DNA above was suspended in 50 μL of H_2_O, heated to 90°C for 30 s and cooled to room temperature over 15 min. The cleavage reaction was initiated by the addition of 50 μL of 2× selection buffer (120 mM HEPES, pH 7.5, 600 mM NaCl, 200 mM KCl, 30 mM MgCl_2_, and 30 mM MnCl_2_), and the reaction mixture was incubated at room temperature for 1 h. This was followed by by the addition of EDTA (0.5 M, pH 8.0) to a final concentration of 30 mM. The DNA in the mixture was precipitated with ethanol and the cleavage product was purified by 10% dPAGE. A DNA marker that has the identical size to the cleavage product was used to guide the separation.

The isolated DNA above was amplified by polymerase chain reaction (PCR) in a volume of 50 μL containing 1× PCR buffer (supplied by the vendor as the 10× buffer), 0.2 mM each of the standard dNTPs, 1.25 U of Tth DNA polymerase, 0.5 μM FP1 (forward primer) and 0.5 μM RP1 (reverse primer). Twelve thermocycles were carried out with the following parameters: 94°C, 30 s (2 min for the first cycle); 52°C, 40 s; 72°C, 45 s. A 1/100-fold dilution of the first PCR product was used for the second PCR using the same condition described above with the exception that RP2 was used instead of RP1. Another PCR2 was performed for internal labeling with ^32^P. This was achieved by following the PCR2 protocol except that 10 μCi of [α-^32^P]dGTP and 0.02 mM non-radioactive dGTP were used to substitute 0.2 mM dGTP. The non-radiolabeled and ^32^P-labeled PCR solutions were combined and the DNA in the mixture was precipitated by ethanol. The desired DNA molecules were purified by 10% dPAGE.

The purified DNA was used to carry out the second selection using the same procedure. 10 selection rounds were conducted with the cleavage time set as follows: 1 h for rounds 1–5, 1 min for rounds 6–7, and 5 s for round 8–10. The cleavage product from round 10 was amplified, cloned and sequenced using a protocol we published previously [41].

### *In vitro* selection with mutant library of LRD-B

Each nucleotide located within the original random-sequence domain of LRD-B was subjected to 30% mutagenesis during chemical synthesis (70% chance for the wild-type nucleotide and 10% each for the remaining three nucleotides). 1000 pmol of the library were used for the reselection experiment following the selection scheme as described above. The cleavage time was set as follows: 1 h for round 1, 10 min for round 2, 1 min for rounds 3 and 4, and 5 s for rounds 5 and 6. The cleavage product from round 6 was cloned and sequenced.

### Kinetic analysis of *cis*-acting constructs

Radioactively labeled candidate DNAzymes were prepared by 5′-phosphorylation with 10 μCi of [γ-^32^P]ATP, ligation to S1 and purification by dPAGE as described above. The cleavage reaction was conducted in 100 μL of 1× SB containing 1 μM ligated construct. 10 μL were withdrawn from the reaction mixture at the following time points: 1, 2, 5, 10, 20, 30, 40, 50, and 60 min. These DNA samples were then subjected to 10% dPAGE for DNA separation. The image of cleaved and uncleaved DNA bands was obtained with a Typhoon Trio+ Imager (GE Healthcare) and the radioactivity of each DNA band was quantified with ImageQuant software (Molecular Dynamics). The percent cleavage of the *cis*-acting DNAzyme was then calculated using Microsoft Excel. First-order rate constants were obtained by curve-fitting using Y = Y_max_ [1−e^−*k*t^], where Y represents the cleavage yield at the time t, Y_max_ is the maximal cleavage yield, and *k* is the first-order rate constant. The kinetic experiments were conducted in triplicates.

### Kinetic analysis of *trans*-acting constructs

Single-turnover conditions: radioactive S1 was prepared through phosphorylation with [γ-^32^P]ATP and T4 PNK, and purified by 10% dPAGE as described above. The cleavage reaction was conducted using the same procedure described for the *cis*-acting constructs except for the use of the S1/LRD-BT1 *trans* construct ([S1] = 10 nM; [LRD-BT1] = 200 nM). The first-order rate constants were obtained through curve-fitting using Y = Y_max_ [1−e^−*k*t^]. The kinetic experiments were performed in triplicates.

Multiple-turnover conditions: The cleavage reaction was performed using 5 nM LRD-BT1. The initial rates were determined for each of the following concentrations of S1: 10, 50, 100, 200, 300, 400, 700, and 1000 nM. The *k*_cat_ and *K*_M_ values were derived using Michaelis-Menten equation.

### Metal-ion dependency

Modified 2× selection buffers were used for this experiment, each of which contained 120 mM HEPES, pH 7.5, 600 mM NaCl, 200 mM KCl, 30 mM M^2+^ (M^2+^ = Ca^2+^, Cd^2+^, Mn^2+^, Ba^2+^, Mg^2+^, Zn^2+^, Co^2+^, Ni^2+^, and Sr^2+^). Each reaction mixture contained 10 nM S1 and 200 nM LRD-BT1 in a reaction volume of 20 μL. After incubation for 30 min, the cleavage reaction mixture was analyzed by dPAGE to obtain percent cleavage.

### Truncation of LRD-B

All truncated sequences were prepared through DNA phosphorylation and ligation with S1, as described above for the full-length LRD-B. The cleavage reaction was conducted in 20 μL of 1× SB containing 1 μM ligated construct. The reaction time was 30 min. The percent cleavage was determined for each construct through dPAGE analysis. The relative activity of each truncation was normalized against the full-length construct by 100 × Y_C_/Y_F_, where Y_C_ and Y_F_ represent the percent cleavage of each truncated sequence and full-length sequence, respectively.

### ATP detection using an aptazyme

The aptazyme detection mixture was prepared by incubating 1 mM ATP with 50 nM S1-Apt1 in 1× SB for 5 min. The reaction was initiated by the addition of 50 nM LRD-Apt1. Aliquots were taken out and quenched with EDTA at the following time points: 1, 2, 5, 10, 20, 30, 60, 120, 180 min. The DNA in each reaction mixture was precipitated with ethanol, separated by 10% dPAGE, and analyzed with a Typhoon Trio+ Imager. The experiments were performed in triplicates.

### DNA methylation

The cleavage reaction was carried out in 400 μL of 1× SB containing 100 pmol of LRD-B. After 30 min, the DNA was precipitated with ethanol and resuspended in 200 μL of H_2_O. The DNA solution was then heated to 90°C for 1 min and cooled to room temperature for 10 min. Subsequently, 200 μL of 0.4% DMS in H_2_O were added and the mixture was incubated at room temperature for 35 min. This methylation sample was denoted “control”. Another sample, denoted “test”, was also produced with 100 pmol of LRD-B, but for this sample, DMS methylation was carried out prior to RNA cleavage. The DNA in both samples was precipitated with ethanol and radioactively labeled at the 5′-end with 10 μCi of [γ-^32^P]ATP and 10 units of PNK. The cleavage fragment was purified by 10% dPAGE, resuspended in 100 μL of 10% piperidine, and heated at 90°C for 30 min. Each mixture was then dried in a vacuum concentrator. The DNA molecules in each sample were separated by 10% dPAGE. The gel image was taken using a Typhoon Trio+ Imager.

### Stability test of D-RNA and L-RNA against RNases

Radioactively labeled S1 and S1-D-RNA (sequence: 5′-ACTCTT CTTAG CT-DrG-TGGTT CGATC AAG) substrates were prepared by 5′-phosphorylation with 10 μCi of [γ-^32^P]ATP. The substrates were then precipitated with ethanol and purified by 10% dPAGE. 50 pmol of S1 or S1-D-RNA were then incubated 1 unit of RNase T1 or RNase I for 15 min at room temperature. Each substrate was also treated with 0.5 M of NaOH for 15 min at 90°C. The DNA molecules in the reaction mixtures were then precipitated with ethanol, separated by 10% dPAGE, and analyzed with a Typhoon Trio+ Imager.

## Supporting Information

S1 Fig Sequencing results with the Round 10 DNA pool. 29 clones were sequenced and they belong to three sequence classes denoted LRD-A (16 copies), LRD-B (9 copies), and LRD-C (4 copies). Note that only the nucleotides located in the random domain of the library L1 are shown (the sequences of the constant primer-binding sites of L1 can be found in Fig 1 of the main manuscript).
(DOCX)

S2 Fig Kinetic analysis of LRD-A, LRD-B, and LRD-C. The *cis*-acting LRD-A, LRD-B and LRD-C were examined for the cleavage activity by measuring % cleavage (Y) at 1, 2, 5, 10, 20, 30, 40, 50, and 60 min. The data were then fitted with the equation Y = Y_max_ [1−e^−*k*t^] to obtain the first-order rate constant *k* and maximal cleavage Y_max_, which are shown in the graph.
(DOCX)

S3 Fig Methylation interference of nucleotides in *cis*-acting LRD-B. Control: cleavage reaction first and DMS treatment second; in this case every G and A residue can be freely methylated. Test: DMS methylation first and cleavage reaction second; in this case the G and A residues that are important to the catalytic function may not be able to accommodate a methyl group. The reduced intensity of the DNA band in the test lane corresponding to A21, A22, and G41, in comparison to the same DNA band in the control lane, reflects strong methylation interference of these nucleotides, suggesting these three residues are important to the function of the DNAzyme. For nucleotide numbering, see Fig 2C of the main manuscript.
(DOCX)

S4 Fig Activation of the S1-Apt1/LRD-Apt1 aptazyme system by ATP. The cleavage of the substrate S1-Apt1 by the DNAzyme LRD-Apt1 in the presence and absence of 1 mM ATP was monitored at the following time points: 1, 2, 5, 10, 20, 30, 60, 120, 180 min. The fraction of the substrate that remained uncleaved is determined and plotted vs. the reaction time.
(DOCX)

S5 Fig Resistance of L-RNA and D-RNA substrates to RNases. RNase T1 or RNase I (1 unit) was with the D-RNA (left) or L-RNA (right) containing substrate for 15 min at room temperature prior to dPAGE analysis. The lane labeled with “NaOH” contained the relevant substrate that was fully hydrolyzed by NaOH (0.5 M of NaOH at 90°C for 15 min).
(DOCX)

## References

[1] Ellington AD, Szostak JW. In vitro selection of RNA molecules that bind specific ligands. Nature. 1990; 346:818–822. 1697402 10.1038/346818a0

[2] Tuerk C, Gold L. Systematic evolution of ligands by exponential enrichment: RNA ligands to bacteriophage T4 DNA polymerase. Science. 1990; 249:505–510. 2200121 10.1126/science.2200121

[3] Breaker RR, Joyce GF. A DNA enzyme that cleaves RNA. Chem Biol. 1994; 1:223–229. 9383394 10.1016/1074-5521(94)90014-0

[4] Schlosser K, Li Y. Biologically inspired synthetic enzymes made from DNA. Chem Biol. 2009; 16:311–322. 10.1016/j.chembiol.2009.01.008 19318212

[5] Baum DA, Silverman SK. Deoxyribozymes: useful DNA catalysts in vitro and in vivo. Cell Mol Life Sci. 2008; 65:2156–2174. 10.1007/s00018-008-8029-y 18373062 PMC7079777

[6] Chandra M, Sachdeva A, Silverman SK. DNA-catalyzed sequence-specific hydrolysis of DNA. Nat Chem Biol. 2009; 5:718–720. 10.1038/nchembio.201 19684594 PMC2746877

[7] Wong OY, Mulcrone AE, Silverman SK. DNA-catalyzed reductive amination. Angew Chem Int Ed. 2011; 50:11679–11684. 10.1002/anie.201104976 21994131 PMC3269127

[8] Chandrasekar J, Silverman SK. Catalytic DNA with phosphatase activity. Proc Natl Acad Sci USA. 2013; 110:5315–5320. 10.1073/pnas.1221946110 23509279 PMC3619321

[9] Parker DJ, Xiao Y, Aguilar JM, Silverman SK. DNA catalysis of a normally disfavored RNA hydrolysis reaction. J Am Chem Soc. 2013; 135:8472–8475. 10.1021/ja4032488 23697866 PMC3702039

[10] Brandsen BM, Velez TE, Sachdeva A, Ibrahim NA, Silverman SK. DNA-catalyzed lysine side chain modification. Angew Chem Int Ed. 2014; 53:9045–9050. 10.1002/anie.201404622 24981820 PMC4136482

[11] Berget SM, Moore C, Sharp PA. Spliced segments at the 5' terminus of adenovirus 2 late mRNA. Proc Natl Acad Sci USA. 1977; 74:3171–3175. 269380 10.1073/pnas.74.8.3171 PMC431482

[12] Chow LT, Roberts JM, Lewis JB, Broker TR. A map of cytoplasmic RNA transcripts from lytic adenovirus type 2, determined by electron microscopy of RNA:DNA hybrids. Cell. 1977; 11:819–836. 890740 10.1016/0092-8674(77)90294-x

[13] Guerrier-Takada C, Gardiner K, Marsh T, Pace N, Altman S. The RNA moiety of ribonuclease P is the catalytic subunit of the enzyme. Cell. 1983; 35:849–857. 6197186 10.1016/0092-8674(83)90117-4

[14] Kruger K, Grabowski PJ, Zaug AJ, Sands J, Gottschling DE, Cech TR. Self-splicing RNA: autoexcision and autocyclization of the ribosomal RNA intervening sequence of Tetrahymena. Cell. 1982; 31:147–157. 6297745 10.1016/0092-8674(82)90414-7

[15] Schlosser K, Li Y. A versatile endoribonuclease mimic made of DNA: characteristics and applications of the 8–17 RNA-cleaving DNAzyme. Chembiochem. 2010; 11:866–879. 10.1002/cbic.200900786 20213779

[16] Silverman SK. In vitro selection, characterization, and application of deoxyribozymes that cleave RNA. Nucleic Acids Res. 2005; 33:6151–6163. 16286368 10.1093/nar/gki930 PMC1283523

[17] Breaker RR, Joyce GF. A DNA enzyme with Mg^2+^-dependent RNA phosphoesterase activity. Chem Biol. 1995; 2:655–660. 9383471 10.1016/1074-5521(95)90028-4

[18] Schlosser K, Gu J, Lam JCF, Li Y. In vitro selection of small RNA-cleaving deoxyribozymes that cleave pyrimidine-pyrimidine junctions. Nucleic Acids Res. 2008; 36:4768–4777. 10.1093/nar/gkn396 18644842 PMC2504313

[19] Li J, Zheng W, Kwon AH, Lu Y. In vitro selection and characterization of a highly efficient Zn(II)-dependent RNA-cleaving deoxyribozyme. Nucleic Acids Res. 2000; 28:481–488. 10606646 10.1093/nar/28.2.481 PMC102519

[20] Liu J, Brown AK, Meng X, Cropek DM, Istok JD, Watson DB, et al A catalytic beacon sensor for uranium with parts-per-trillion sensitivity and millionfold selectivity. Proc Natl Acad Sci USA. 2007; 104:2056–2061. 17284609 10.1073/pnas.0607875104 PMC1892917

[21] Huang PJ, Vazin M, Liu J. In vitro selection of a new lanthanide-dependent DNAzyme for ratiometric sensing lanthanides. Anal Chem. 2014; 86:9993–9999. 10.1021/ac5029962 25199650

[22] Li J, Lu Y. A highly sensitive and selective catalytic DNA biosensor for lead ions. J Am Chem Soc. 2000; 122:10466–10467.

[23] Lu LM, Zhang XB, Kong RM, Yang B, Tan W. A ligation-triggered DNAzyme cascade for amplified fluorescence detection of biological small molecules with zero-background signal. J Am Chem Soc. 2011; 133:11686–11691. 10.1021/ja203693b 21662240 PMC5512710

[24] Ali MM, Aguirre SD, Lazim H, Li Y. Fluorogenic DNAzyme probes as bacterial indicators. Angew Chem Int Ed. 2011; 50:3751–3754. 10.1002/anie.201100477 21412961

[25] Zhang XB, Kong RM, Lu Y. Metal ion sensors based on DNAzymes and related DNA molecules. Annu Rev Anal Chem. 2011; 4:105–128. 10.1146/annurev.anchem.111808.073617 21370984 PMC3119750

[26] Liu J, Lu Y. A colorimetric lead biosensor using DNAzyme-directed assembly of gold nanoparticles. J Am Chem Soc. 2003; 125:6642–6643. 12769568 10.1021/ja034775u

[27] Liu J, Lu Y. Stimuli-responsive disassembly of nanoparticle aggregates for light-up colorimetric sensing. J Am Chem Soc. 2005; 127:12677–12683. 16144417 10.1021/ja053567u

[28] Zhao W, Lam JC, Chiuman W, Brook MA, Li Y. Enzymatic cleavage of nucleic acids on gold nanoparticles: a generic platform for facile colorimetric biosensors. Small. 2008; 4:810–816. 10.1002/smll.200700757 18537135

[29] Xiao Y, Qu X, Plaxco KW, Heeger AJ. Label-free electrochemical detection of DNA in blood serum via target-induced resolution of an electrode-bound DNA pseudoknot. J Am Chem Soc. 2007; 129:11896–11897. 17850085 10.1021/ja074218y

[30] Chiuman W, Li Y. Simple fluorescent sensors engineered with catalytic DNA 'MgZ' based on a non-classic allosteric design. PLoS One. 2007; 2:e1224 18030352 10.1371/journal.pone.0001224 PMC2077808

[31] Mei SH, Liu Z, Brennan JD, Li Y. An efficient RNA-cleaving DNA enzyme that synchronizes catalysis with fluorescence signaling. J Am Chem Soc. 2003; 125:412–420. 12517153 10.1021/ja0281232

[32] Yang X, Xu J, Tang X, Liu H, Tian D. A novel electrochemical DNAzyme sensor for the amplified detection of Pb^2+^ ions. Chem Commun. 2010; 46:3107–3109. 10.1039/c002137g 20361096

[33] Tram K, Kanda P, Salena BJ, Huan S, Li Y. Translating bacterial detection by DNAzymes into a litmus test. Angew Chem Int Ed. 2014; 53:12799–12802. 10.1002/anie.201407021 25213464

[34] He S, Qu L, Shen Z, Tan Y, Zeng M, Liu F, et al Highly Specific Recognition of Breast Tumors by an RNA-Cleaving Fluorogenic DNAzyme Probe. Anal Chem. 2015; 87:569–577. 10.1021/ac5031557 25479319

[35] Klussmann S, Nolte A, Bald R, Erdmann VA, Furste JP. Mirror-image RNA that binds D-adenosine. Nat Biotechnol. 1996; 14:1112–1115. 9631061 10.1038/nbt0996-1112

[36] Ashley GW. Modeling, synthesis, and hybridization properties of (L)-ribonucleic acid. J Am Chem Soc. 1992; 114:9731–9736.

[37] Garbesi A, Capobianco ML, Colonna FP, Tondelli L, Arcamone F, Manzini G, et al L-DNAs as potential antimessenger oligonucleotides: a reassessment. Nucleic Acids Res. 1993; 21:4159–4165. 8414968 10.1093/nar/21.18.4159 PMC310044

[38] Sczepanski JT, Joyce GF. Binding of a structured D-RNA molecule by an L-RNA aptamer. J Am Chem Soc. 2013; 135:13290–13293. 10.1021/ja406634g 23977945 PMC3804424

[39] Ordoukhanian P, Joyce GF. RNA-cleaving DNA enzymes with altered regio- or enantioselectivity. J Am Chem Soc. 2002; 124:12499–12506. 12381192 10.1021/ja027467p

[40] Achenbach JC, Jeffries GA, McManus SA, Billen LP, Li Y. Secondary-structure characterization of two proficient kinase deoxyribozymes. Biochemistry. 2005; 44:3765–3774. 15751953 10.1021/bi0483054

[41] Wang W, Billen LP, Li Y. Sequence diversity, metal specificity, and catalytic proficiency of metal-dependent phosphorylating DNA enzymes. Chem Biol. 2002; 9:507–517. 11983339 10.1016/s1074-5521(02)00127-8

[42] Liu Z, Mei SH, Brennan JD, Li Y. Assemblage of signaling DNA enzymes with intriguing metal-ion specificities and pH dependences. J Am Chem Soc. 2003; 125:7539–7545. 12812493 10.1021/ja035208+

[43] Cruz RP, Withers JB, Li Y. Dinucleotide junction cleavage versatility of 8–17 deoxyribozyme. Chem Biol. 2004; 11:57–67. 15112995 10.1016/j.chembiol.2003.12.012

[44] Xiao Y, Allen EC, Silverman SK. Merely two mutations switch a DNA-hydrolyzing deoxyribozyme from heterobimetallic (Zn^2+^/Mn^2+^) to monometallic (Zn^2+^-only) behavior. Chem Commun. 2011; 47:1749–1751. 10.1039/c0cc04575f 21125108 PMC3269124

[45] Tang J, Breaker RR. Rational design of allosteric ribozymes. Chem Biol. 1997; 4:453–459. 9224568 10.1016/s1074-5521(97)90197-6

[46] Tang J, Breaker RR. Mechanism for allosteric inhibition of an ATP-sensitive ribozyme. Nucleic Acids Res. 1998; 26:4214–4221. 9722642 10.1093/nar/26.18.4214 PMC147823

[47] Wang DY, Lai BH, Sen D. A general strategy for effector-mediated control of RNA-cleaving ribozymes and DNA enzymes. J Mol Biol. 2002; 318:33–43. 12054766 10.1016/S0022-2836(02)00046-3

[48] Huizenga DE, Szostak JW. A DNA aptamer that binds adenosine and ATP. Biochemistry. 1995; 34:656–665. 7819261 10.1021/bi00002a033

[49] Santoro SW, Joyce GF. A general purpose RNA-cleaving DNA enzyme. Proc Natl Acad Sci USA. 1997; 94:4262–4266. 9113977 10.1073/pnas.94.9.4262 PMC20710

