# Supplementary material for: An Efficient Catalytic DNA that Cleaves L-RNA
Source: PLoS One. 2015 May 6;10(5):e0126402. doi: 10.1371/journal.pone.0126402 (PMC4422682; doi:10.1371/journal.pone.0126402)
Supplement: S1 Fig — 29 clones were sequenced and they belong to three sequence classes denoted LRD-A (16 copies), LRD-B (9 copies), and LRD-C (4 copies). Note that only the nucleotides located in the random domain of the library L1 are shown (the sequences of the constant primer-binding sites of L1 can be found in Fig 1 of the main manuscript). (DOCX) [file pone.0126402.s001.docx]

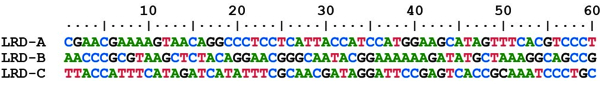


**S1 Fig**. **Sequencing results with the round 10 DNA pool.** 29 clones were sequenced and they belong to three sequence classes denoted LRD-A (16 copies), LRD-B (9 copies), and LRD-C (4 copies). Note that only the nucleotides located in the random domain of the library L1 are shown (the sequences of the constant primer-binding sites of L1 can be found in Fig. 1 of the main manuscript).
