# Supplementary material for: An Efficient Catalytic DNA that Cleaves L-RNA
Source: PLoS One. 2015 May 6;10(5):e0126402. doi: 10.1371/journal.pone.0126402 (PMC4422682; doi:10.1371/journal.pone.0126402)
Supplement: S2 Fig — The cis-acting LRD-A, LRD-B and LRD-C were examined for the cleavage activity by measuring % cleavage (Y) at 1, 2, 5, 10, 20, 30, 40, 50, and 60 min. The data were then fitted with the equation Y = Ymax [1−e−kt] to obtain the first-order rate constant k and maximal cleavage Ymax, which are shown in the graph. (DOCX) [file pone.0126402.s002.docx]

**
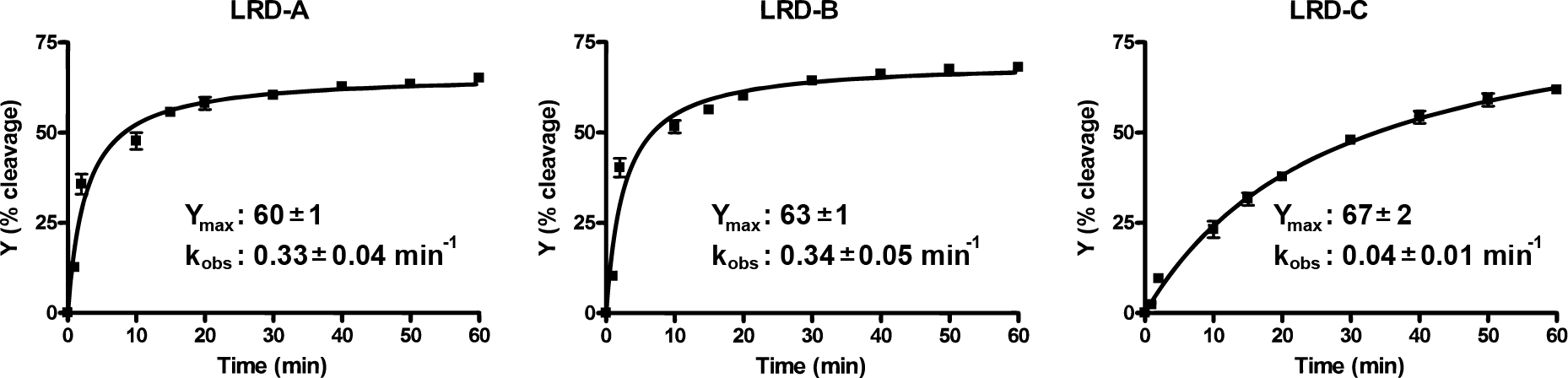
**

**S2 Fig**. **Kinetic analysis of LRD-A, LRD-B, and LRD-C.** The *cis*-acting LRD-A, LRD-B and LRD-C were examined for the cleavage activity by measuring % cleavage (Y) at 1, 2, 5, 10, 20, 30, 40, 50, and 60 min. The data were then fitted with the equation Y = Y_max_ [1−e^−^*^k^*^t^] to obtain the first-order rate constant *k* and maximal cleavage Y_max_, which are shown in the graph.
