# Supplementary material for: An Efficient Catalytic DNA that Cleaves L-RNA
Source: PLoS One. 2015 May 6;10(5):e0126402. doi: 10.1371/journal.pone.0126402 (PMC4422682; doi:10.1371/journal.pone.0126402)
Supplement: S3 Fig — Control: cleavage reaction first and DMS treatment second; in this case every G and A residue can be freely methylated. Test: DMS methylation first and cleavage reaction second; in this case the G and A residues that are important to the catalytic function may not be able to accommodate a methyl group. The reduced intensity of the DNA band in the test lane corresponding to A21, A22, and G41, in comparison to the same DNA band in the control lane, reflects strong methylation interference of these nucleotides, suggesting these three residues are important to the function of the DNAzyme. For nucleotide numbering, see Fig 2C of the main manuscript. (DOCX) [file pone.0126402.s003.docx]

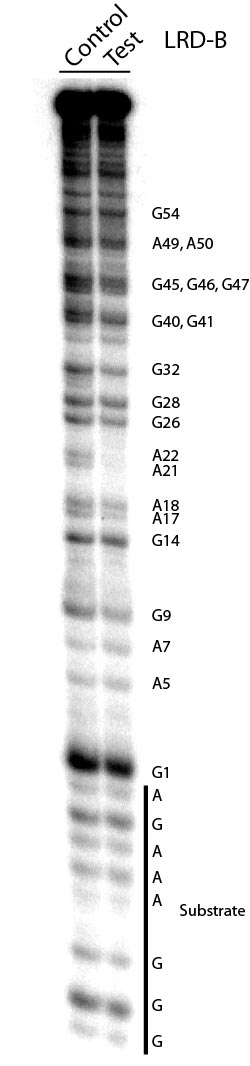


**S3 Fig**. **Methylation interference of nucleotides in *cis*-acting LRD-B.** Control: cleavage reaction first and DMS treatment second; in this case every G and A residue can be freely methylated. Test: DMS methylation first and cleavage reaction second; in this case the G and A residues that are important to the catalytic function may not be able to accommodate a methyl group. The reduced intensity of the DNA band in the test lane corresponding to A21, A22, and G41, in comparison to the same DNA band in the control lane, reflects strong methylation interference of these nucleotides, suggesting these three residues are important to the function of the DNAzyme. For nucleotide numbering, see Fig. 2C of the main manuscript.
