# Supplementary material for: An Efficient Catalytic DNA that Cleaves L-RNA
Source: PLoS One. 2015 May 6;10(5):e0126402. doi: 10.1371/journal.pone.0126402 (PMC4422682; doi:10.1371/journal.pone.0126402)
Supplement: S4 Fig — The cleavage of the substrate S1-Apt1 by the DNAzyme LRD-Apt1 in the presence and absence of 1 mM ATP was monitored at the following time points: 1, 2, 5, 10, 20, 30, 60, 120, 180 min. The fraction of the substrate that remained uncleaved is determined and plotted vs. the reaction time. (DOCX) [file pone.0126402.s004.docx]

**S4 Fig**. **Activation of the** **S1-Apt1/LRD-Apt1 aptazyme system by ATP.** The cleavage of the substrate S1-Apt1 by the DNAzyme LRD-Apt1 in the presence and absence of 1 mM ATP was monitored at at the following time points: 1, 2, 5, 10, 20, 30, 60, 120, 180 min. The fraction of the substrate that remained uncleaved is determined and plotted vs. the reaction time.
