# Supplementary material for: An Efficient Catalytic DNA that Cleaves L-RNA
Source: PLoS One. 2015 May 6;10(5):e0126402. doi: 10.1371/journal.pone.0126402 (PMC4422682; doi:10.1371/journal.pone.0126402)
Supplement: S5 Fig — RNase T1 or RNase I (1 unit) was with the D-RNA (left) or L-RNA (right) containing substrate for 15 min at room temperature prior to dPAGE analysis. The lane labeled with “NaOH” contained the relevant substrate that was fully hydrolyzed by NaOH (0.5 M of NaOH at 90°C for 15 min). (DOCX) [file pone.0126402.s005.docx]

**
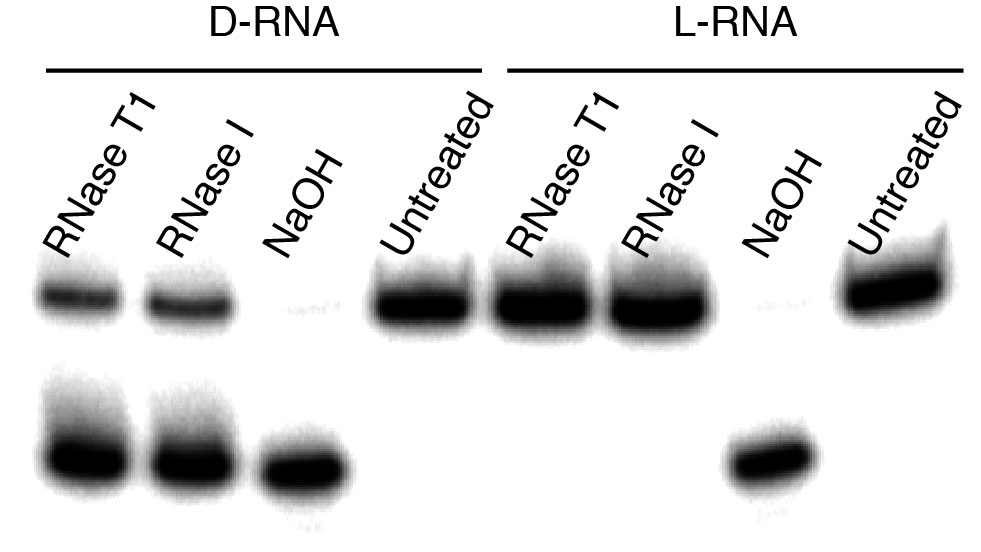
**

**S5 Fig**. **Resistance of L-RNA and D-RNA substrates to RNases.** RNase T1 or RNase I (1 unit) was with the D-RNA (left) or L-RNA (right) containing substrate for 15 min at room temperature prior to dPAGE analysis. The lane labeled with “NaOH” contained the relevant substrate that was fully hydrolyzed by NaOH (0.5 M of NaOH at 90°C for 15 min).
